# Free-Standing GaMnAs Nanomachined Sheets for van der Pauw Magnetotransport Measurements

**DOI:** 10.3390/mi7120223

**Published:** 2016-12-09

**Authors:** Jae-Hyun Lee, Seondo Park, Chanuk Yang, Hyung Kook Choi, Myung Rae Cho, Sung Un Cho, Yun Daniel Park

**Affiliations:** 1 Department of Physics & Astronomy and Institute of Applied Physics, Seoul National University, 1 Gwanak-ro, Gwanak-gu, Seoul 08826, Korea; bluej11@snu.ac.kr (J.-H.L.); sheldon17@snu.ac.kr (S.P.); ycu15@snu.ac.kr (C.Y.); inhuman7@snu.ac.kr (M.R.C.); nebula45@snu.ac.kr (S.U.C.); 2 Department of Physics, Chonbuk National University, 567 Baekje-daero, Deokjin-gu, Jeonju-si, Jeollabuk-do 54896, Korea; hkchoi@jbnu.ac.kr

**Keywords:** free-standing structure, magnetotranport, magnetic anisotropy

## Abstract

We report on the realization of free-standing GaMnAs epilayer sheets using nanomachining techniques. By optimizing the growth conditions of the sacrificial Al_0.75_Ga_0.25_As layer, free-standing metallic GaMnAs (with ~6% Mn) microsheets (with *T_C_* ~85 K) with integrated electrical probes are realized for magnetotransport measurements in the van der Pauw geometry. GaMnAs epilayer needs to be physically isolated to avoid buckling effects stemming from the release of lattice mismatch strain during the removal of the AlGaAs sacrificial layer. From finite element analysis, symmetrically placed and serpentine-shaped electrical leads induce minimal thermal stress at low temperatures. From magnetotransport measurements, changes in magnetic anisotropy are readily observed.

## 1. Introduction

Since the first observation of ferromagnetic ordering in highly Mn-doped GaAs two decades ago [1], the ferromagnetic diluted magnetic semiconductor (DMS) GaMnAs has been an instrumental “test bed” for spintronics and magnetic systems with strong spin-orbit interactions [2,3]. Carrier-mediated ferromagnetic ordering in DMS systems can be readily observed as a dependence of the magnetic ordering temperature (*T_C_*) on the hole carrier concentration as well as through the novel control of ferromagnetism by electric fields [4,5] and light [6]. Such novel demonstrations are facilitated by magnetotransport measurements, such as measurements of the temperature dependence of the resistivity, anisotropic magnetoresistance (AMR) measurements, and measurements of the anomalous Hall effect (AHE). In metallic GaMnAs samples, an anomaly in the temperature dependence of the resistivity is seen near *T_C_* [7], AMR measurements, particularly in the planar Hall effect geometry, have been instrumental in studying magnetic anisotropy [8]. Since the advent of the low-temperature molecular beam epitaxy (LT-MBE) of GaMnAs, it has been well established and documented that the choice of the underlying substrate and the resulting strain from lattice mismatch, either compressive or tensile, exerts a large influence on the magnetization direction [9]. Demonstrations of the static control of the magnetic anisotropy in GaMnAs by means of post-growth patterning via strain relaxation [10], domain wall motion [11], and reversible magnetization induced by relativistic effects [12] may offer new avenues for the development of GaMnAs-based spintronic devices and applications. In magnetic systems with strong spin-orbit interactions, magnetocrystalline anisotropy, which is readily evident in AMR and planar Hall effect measurements, is generally a direct manifestation of this phenomenon. An understanding of the AHE and its intrinsic origins in GaMnAs [13] has led to the development of a directly related spin Hall effect and other related relativistic effects, such as spin transfer torque. AHE measurements, along with the scaling relationship between the longitudinal conductivity and the Hall conductivity, demonstrate the intrinsic nature of GaMnAs [14] and have been shown to be sensitive to secondary phases [15]. Because relativistic spin-orbit interactions are highly sensitive to strain, a dynamic means of varying the strain in GaMnAs may lead to a better understanding of this behavior and serve as an avenue for adding new functionalities, stemming from mechanical degrees of freedom, to develop novel GaMnAs-based spintronic devices [16].

Here, we demonstrate the first steps of incorporating mechanical functionalities into GaMnAs-based spintronic devices by utilizing a surface nanomachining process that is commonly applied in nanoelectromechanical systems (NEMS) research [17] to realize free-standing GaMnAs epilayer sheet structures with incorporated metallic electrical leads. A key process in nanomachining is selective wet etching between a layer of interest and the sacrificial layer. From research and development on III–V high-electron-mobility transistors (HEMTs) and light-emitting diodes (LEDs), processes for highly selective etching between GaAs and various possible sacrificial layers are well known [18]. In the literature on the LT-MBE growth of GaMnAs, buffer layers of GaAs, AlGaAs, and InGaAs dominate. For the most extensively studied GaMnAs/GaAs system, the GaMnAs layer is usually strained because of substitutional Mn and As antisites originating from growth at low temperatures, and selective etching is difficult. GaMnAs/InGaAs structures have been studied in some depth. Because the lattice constant of InGaAs is larger than that of GaMnAs, a tensile strain results in a magnetization in the GaMnAs layer in the direction perpendicular to the plane. However, under optimal conditions, there is finite selectivity between GaAs and In_1−*x*_Ga*_x_*As, whereas for GaAs/Al_1−*x*_Ga*_x_*As and GaAs/In_1−*x*_Ga*_x_*P, nearly infinite selectivity exists. In particular, GaMnAs/AlAs heterostructures have been studied in some depth to realize GaMnAs-based tunneling anisotropic magnetoresistance (TAMR) devices [19,20]. GaMnAs/InGaP can be tuned to have nearly zero lattice mismatch, which is ideal for realizing nanomechanical structures [21], but this requires the regrowth of high-quality GaMnAs on a prepared InGaP surface, which has been found to be difficult. As a compromise between the realization of a high-quality metallic GaMnAs epilayer and a high selectivity of the underlying sacrificial/buffer layer, we choose a GaMnAs/GaAs/Al_0.75_Ga_0.25_As heterostructure. Pseudomorphic growth imposes a lateral strain on the GaMnAs, the release of which may induce buckling, requiring the incorporation of separate metallic leads. Using the incorporated electrical leads, magnetotransport measurements can be performed to determine the magnetic properties of the free-standing GaMnAs layer.

## 2. Materials and Methods

**Figure 1:**
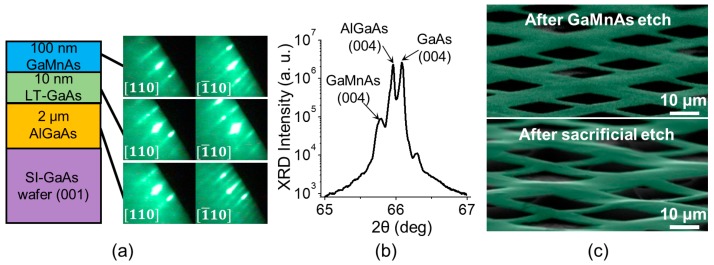
(**a**) GaMnAs/LT-GaAs/AlGaAs/SI-GaAs structure and corresponding RHEED images. (**b**) HR-XRD *θ–*2*θ* measurement of the sample. (**c**) Scanning electron microscope (SEM) images of GaMnAs crosses after the GaMnAs etch (**top**) and after the sacrificial etch (**bottom**), which show buckling of the GaMnAs epilayer after the removal of the underlying AlGaAs sacrificial layer. GaMnAs layer is distinguished with added false color (green).

To realize free-standing metallic GaMnAs epilayers, we grew GaMnAs (100 nm)/GaAs (10 nm)/AlGaAs (2000 nm) on a SI-GaAs (001) substrate (Figure 1a). After the preparation of the substrate surface, an Al_1−*x*_Ga*_x_*As layer was grown at a substrate temperature (*T_S_*) of 600 °C. Under a constant As flux, *T_S_* was lowered to 200 °C for the growth of a thin LT-GaAs buffer layer; then, an Mn flux was introduced for the growth of the final GaMnAs layer. The LT-MBE growth of GaMnAs has been described in detail elsewhere [14]. Throughout the growth, RHEED monitoring was conducted to ensure high-quality growth. After growth, an high resolution x-ray diffraction (HR-XRD) *θ–*2*θ* measurement revealed an Mn concentration of ~6% in the GaMnAs epilayer and an Al concentration of 75% in the AlGaAs sacrificial/buffer layer (Figure 1b). After growth, the sample was annealed at 200 °C for 1 h under a dry flow of N_2_. The resulting lattice mismatch between the GaMnAs epilayer and the AlGaAs sacrificial layer corresponded to a compressive strain of ~0.2%. Structural beams buckle because of compressive stresses applied along their axes. The force required is often called the critical load (or the Euler load). From mechanical beam theory, this critical load is $\sigma_{c}=n^{2}\pi^{2}EI/L^{2}$, where *n* is the buckling mode, *E* is the Young’s modulus (here approximated for bulk GaAs), *I* is the beam’s moment of inertial about the neutral axis, and *L* is the beam length. For reasonable geometries, if the lattice mismatch strain were to be released during the removal of the sacrificial layer, we would expect the GaMnAs layer to buckle. To illustrate such buckling, we repeatedly patterned cross-like structures, representing GaMnAs Hall crosses, using standard e-beam lithography techniques. We patterned the GaMnAs crosses by selectively removing exposed GaMnAs layers via etching with citric acid [22]. After the removal of the e-beam resist, the underlying AlGaAs sacrificial layer was selectively etched with 10% HF [23,24]. After the etching of the sacrificial layer, the sample was dried using a critical point dryer (CPD). As seen in Figure 1c, the free-standing GaMnAs epilayers buckle.

## 3. Results and Discussion

**Figure 2:**
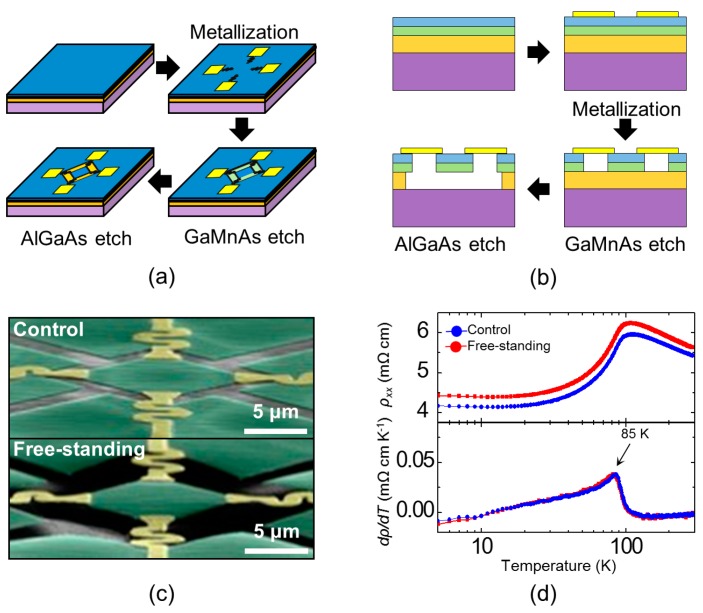
(**a**) Schematic illustration of the fabrication of a free-standing GaMnAs microsheet with integrated electrical leads suitable for use in van der Pauw measurements. (**b**) Cross-sectional schematic view of fabrication process. (**c**) SEM image of control (**top**) and free-standing (**bottom**) van der Pauw structures with added false color to distinguish the GaMnAs layer (green) and electrical leads (gold). (**d**) Temperature-dependent resistivity (ρ*_xx_* vs. *T*) and the corresponding differential resistivity (*d*ρ*_xx_*/*dT*).

To avoid buckling, we patterned and physically isolated the GaMnAs epilayers. The fabrication process for a 10 µm × 10 µm GaMnAs sheet suitable for use in the van der Pauw geometry is outlined in Figure 2a,b. First, by means of e-beam lithography and e-beam evaporation, electrical leads of Au (90 nm)/Cr (10 nm) were fashioned, which also served to physically anchor the resulting GaMnAs microsheet. Van der Pauw structures were defined via e-beam lithography and a citric acid/hydrogen peroxide etch at a volume ratio of 7:2 (etch rate: ~200 nm/min). During the etching of the GaMnAs, the etching time and the lithographic patterns were deliberately controlled to ensure that the GaMnAs below the metallic electrical leads was completely removed and the GaMnAs structure was physically isolated. Then, the sample was subjected to a selective sacrificial etch using 10% HF to remove the sacrificial AlGaAs. Finally, the critical point drying method was applied. In addition to the free-standing GaMnAs structures, control structures of GaMnAs/AlGaAs were also prepared using the same nanomachining process, protected with e-beam resist during the AlGaAs etch. Scanning electron microscope (SEM) images of both control (unsuspended) and free-standing (suspended) van der Pauw structures with dimensions of 10 µm × 10 µm are presented in Figure 2c.

To demonstrate the quality of the free-standing GaMnAs sheets, the temperature dependence of the resistivity was plotted for both the free-standing GaMnAs and the control GaMnAs/AlGaAs structures (Figure 2d). All magnetotransport measurements were performed in a commercially available closed-cycle magnetocryostat (IceOxford ^DRY^ICE^4TL^, ICE Oxford Ltd., Witney, UK) using the standard AC lock-in transport technique (*I* = 0.1 µA at 17 Hz and 23 Hz). Both structures exhibited clear metallic behavior and a temperature-dependence anomaly corresponding to *T_C_* ~85 K. For the free-standing GaMnAs, ρ*_xx_* < 10 mΩ·cm; this resistivity is consistent with GaMnAs exhibiting an intrinsic AHE [14]. Although the free-standing GaMnAs sheets had an increased free surface area (from which the interstitial Mn may diffuse), we did not perform post-processing low-temperature annealing, which may increase *T_C_* [25]. The fact that the resistivity of the free-standing GaMnAs was higher than that of the control suggests increased scattering from these free surfaces.

**Figure 3:**
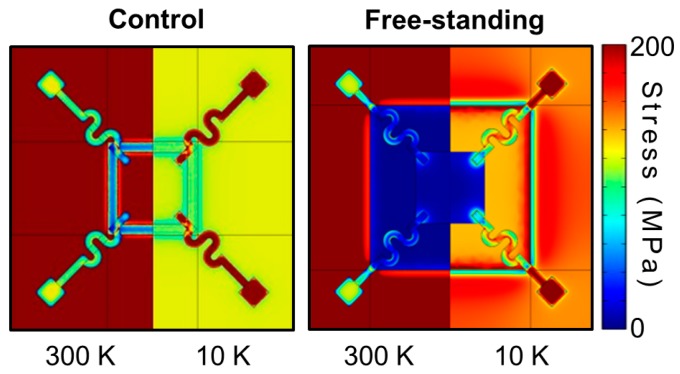
Finite-element analysis mapping the calculated stress distributions for the control and free-standing GaMnAs microsheets for *T* = 300 K and 10 K.

Although the electrical leads were symmetrically patterned with a serpentine geometry to minimize any thermally induced strain, because of the large difference between the coefficient of thermal expansion of GaMnAs and that of the metallic electrical leads, we could not fully eliminate such thermoelastic/piezoresistive effects, which may have served as a source of the slight discrepancy between the temperature dependences of the resistivities of the two structures. We attempted to quantify the thermally induced strain using a commercially available finite-element analysis package (COMSOL Multiphysics 4.3b, COMSOL Inc., Burlington, VT, USA). Figure 3 summarizes the results of the finite-element analysis by mapping the expected stress for the control GaMnAs/GaAs/AlGaAs structure and the free-standing GaMnAs microsheet structure anchored by serpentine-shaped metallic leads at the corners for 300 K and 10 K. At *T* = 10 K, much of the stress is limited to the sections with the leads, as expected. On the free-standing GaMnAs sheet, the stress is nearly uniform, and areas of high stress are limited to the regions where the metallic leads clamp the corners of the sheet (with a maximum stress value of ~400 MPa, which rapidly falls to <100 MPa within 100 nm from the edge). Using the Young’s modulus of GaAs, which is 86 GPa, we can estimate the effective strains to be less than 0.001 at 10 K, which we expect to have minimal effects on ρ*_xx_* and ρ*_xy_* [26].

**Figure 4:**
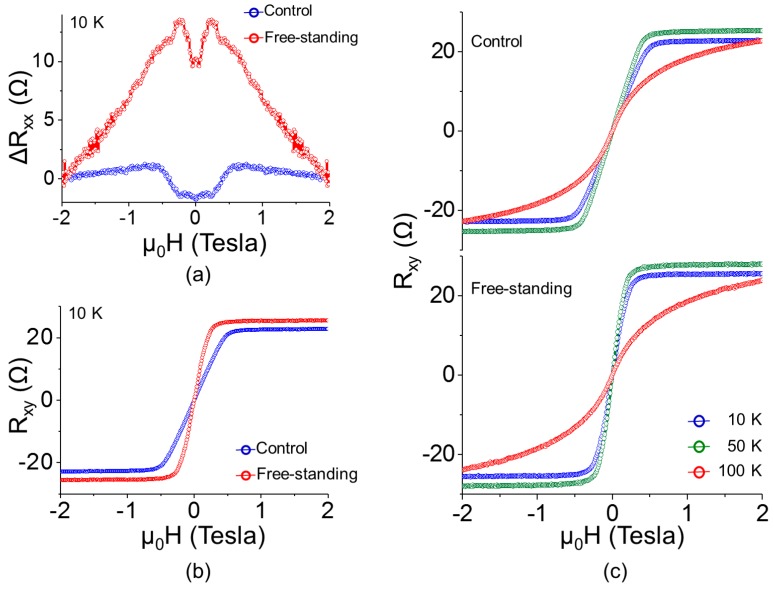
(**a**) Longitudinal Hall resistance as a function of the applied magnetic field (Δ*R_xx_* vs. *H*) at *T* = 10 K. (**b**) Transverse Hall resistance as a function of the applied magnetic field (*R_xy_* vs. *H*) at *T* = 10 K. (**c**) Field-dependent transverse Hall resistance (*R_xy_* vs. *H*) at various temperatures.

Magnetotransport measurements were performed to simultaneously measure ρ*_xx_* and ρ*_xy_* with an applied magnetic field perpendicular to the free-standing GaMnAs microsheet (*H* < ±9 T) using the AC lock-in measurement technique (*I* = 0.1 µA at 17 Hz and 23 Hz). Figure 4a plots the longitudinal resistance as a function of the applied magnetic field at *T* = 10 K. The low-field response (*H* < ±1 T) shows similar absolute resistance changes for both the free-standing GaMnAs and the control. The switching characteristics, however, show marked differences: the magnetization of the free-standing GaMnAs flips at much lower fields. Figure 4b plots the transverse resistance as a function of the applied magnetic field at *T* = 10 K. As implied by the AMR response presented in Figure 4a, the Hall response also suggests that the easy magnetization direction is more in-plane-like for the control sample and more out-of-plane-like for the free-standing GaMnAs microsheet. Beyond GaMnAs, similar effects have been reported in metallic magnetic thin films in which the magnetic anisotropy was varied by applying strain to a flexible substrate [27]. Such changes in the GaMnAs magnetic anisotropy warrant a further detailed study by means of planar Hall effect measurements. Figure 4c plots the transverse resistance as a function of the applied field at temperature of 10 K, 50 K, and 100 K. At temperatures below *T_C_*, the Hall response from the free-standing sample suggests a stronger Hall effect contribution from spontaneous magnetization, which is ascribed to the more out-of-plane-like easy magnetization direction. For both samples, a remnant AHE is readily seen above *T_C_*. This remnant AHE is due to residual spontaneous magnetization, which is ascribed to the second-order transition (or continuous transition) from ferromagnetism to paramagnetism.

## 4. Conclusions

We applied nanomachining techniques to realize free-standing GaMnAs microsheets suitable for use in van der Pauw magnetotransport measurements. By optimizing the growth conditions of the sacrificial AlGaAs layer, high-quality metallic GaMnAs structures were realized. With a delicate modification of the fabrication process and the incorporation of metallic electrical leads, free-standing GaMnAs structures were achieved, avoiding buckling during removal of the sacrificial layer. Free-standing GaMnAs structure, with significantly reduced strain from lattice mismatch and thermal stress, exhibited more out-of-plane-like magnetic anisotropy compare to controlled structures. By modifying the details of the processing steps, various free-standing GaMnAs structures are possible as well as the incorporation of integrated electrical gate structures to controllably and dynamically actuate the free-standing GaMnAs structures by means of electrostatic forces and strain, thereby allowing for the integration of a mechanical degree of freedom into GaMnAs-based spintronic devices.

Realization of free-standing GaMnAs micro-sheets of high-quality for magnetotransport measurements allows for further development of free-standing GaMnAs micro-Hall bar structures. From such Hall bar structures, further magnetotransport measurements (both in standard Hall and planar Hall geometry) would lead to a better understanding of substrate effects on the magneto-anisotropy of the resulting GaMnAs epilayer. Furthermore, with further device engineering, electrostatic gates could be incorporated, which may be used to actuate the buckling process. Controlled buckling would result in a means to dynamically vary the strain on the GaMnAs, possibly allowing for a GaMnAs bit based on mechanical states.
